# Single Exposure to Low-Intensity Focused Ultrasound Causes Biphasic Opening of the Blood-Brain Barrier Through Secondary Mechanisms

**DOI:** 10.3390/pharmaceutics17010075

**Published:** 2025-01-08

**Authors:** Tasneem A. Arsiwala, Kathryn E. Blethen, Cullen P. Wolford, Geoffrey L. Pecar, Dhruvi M. Panchal, Brooke N. Kielkowski, Peng Wang, Manish Ranjan, Jeffrey S. Carpenter, Victor Finomore, Ali Rezai, Paul R. Lockman

**Affiliations:** 1 Department of Pharmaceutical Sciences, West Virginia University School of Pharmacy, Morgantown, WV 26505, USAgp00021@mix.wvu.edu (G.L.P.);; 2 Department of Neuroscience, West Virginia University School of Medicine, Rockefeller Neuroscience Institute, Morgantown, WV 26505, USA; 3 Department of Neuroradiology, West Virginia University School of Medicine, Rockefeller Neuroscience Institute, Morgantown, WV 26505, USA; 4 Department of Neurosurgery, West Virginia University School of Medicine, Rockefeller Neuroscience Institute, Morgantown, WV 26505, USA

**Keywords:** blood–brain barrier (BBB) disruption, low-intensity focused ultrasound (LiFUS), P-glycoprotein (P-gp), vascular permeability, neuroinflammation

## Abstract

**Background/Objective:** The blood–brain barrier (BBB) is selectively permeable, but it also poses significant challenges for treating CNS diseases. Low-intensity focused ultrasound (LiFUS), paired with microbubbles is a promising, non-invasive technique for transiently opening the BBB, allowing enhanced drug delivery to the central nervous system (CNS). However, the downstream physiological effects following BBB opening, particularly secondary responses, are not well understood. This study aimed to characterize the time-dependent changes in BBB permeability, transporter function, and inflammatory responses in both sonicated and non-sonicated brain tissues following LiFUS treatment. **Methods:** We employed in situ brain perfusion to assess alterations in BBB integrity and transporter function, as well as multiplex cytokine analysis to quantify the inflammatory response. **Results:** Our findings show that LiFUS significantly increased vascular volume and glucose uptake, with reduced P-gp function in brain tissues six hours post treatment, indicating biphasic BBB disruption. Additionally, elevated levels of pro-inflammatory cytokines, including TNF-α and IL-6, were observed in both sonicated and non-sonicated regions. A comparative analysis between wild-type and immunodeficient mice revealed distinct patterns of cytokine release, with immunodeficient mice showing lower serum concentrations of IFN-γ and TNF-α, highlighting the potential impact of immune status on the inflammatory response to LiFUS. **Conclusions:** This study provides new insights into the biphasic nature of LiFUS-induced BBB disruption, emphasizing the importance of understanding the timing and extent of secondary physiological changes.

## 1. Introduction

One of the fundamental functions of the blood–brain barrier (BBB) is to regulate the movement of essential metabolites within the central nervous system (CNS) [1]. The BBB is made up of a continuous layer of endothelial cells that are circumferentially joined by tight junctions [2]. BBB integrity is critical for protection of the CNS in both healthy and pathologic states and is maintained by interactions between endothelial cells, pericytes, microglia, astrocytes, and neurons [2]. Unrestricted interlinkage and communication between these diverse cell types is critical for the proper regulation and operation of the BBB. Mechanisms of transport across the BBB include passive diffusion, paracellular transport dependent on interactions with junctional adhesion molecules and accessory proteins, and active transport mediated by luminal transporter and carrier proteins [3]. While the semi-permeable nature of the BBB is crucial in a healthy physiological state, therapeutic delivery to the brain can be highly limited.

The identification and characterization of driving oncogenes has enabled substantial improvements in the control of many cancers, but the efficacy of targeted therapies for the treatment of CNS tumors remains limited [4]. Efforts to overcome the limited brain distribution of therapeutics have historically paired targeted therapies with local delivery strategies, but these have achieved only limited clinical success [3,5]. Emerging methods designed to achieve therapeutically effective drug concentrations within the CNS are focused on transient BBB opening through disruptive techniques such as focused ultrasound, radiation, or osmotic reagents [6]. Microbubble-assisted low-intensity transcranial focused ultrasound (LiFUS) is a technique for the non-invasive, selective, and reversible breakdown of the BBB [7]. LiFUS has demonstrated efficacy in enhancing therapeutic delivery to the CNS in both preclinical and clinical studies [7,8,9]. Existing data suggest that LiFUS can temporarily open the BBB without significantly damaging endothelial cells, astrocytes, or neurons [10,11]. The mechanism by which LiFUS facilitates the accumulation of ordinarily BBB-impermeable therapeutic agents within the CNS involves the exertion of physical force on the tight junctions and transcellular spaces of the protective endothelial barrier [7].

Current research on LiFUS-assisted BBB disruption is primarily centered on technical and safety issues related to the delivery of small and large molecules. Numerous studies have investigated the relationship between BBB function and the FUS parameters used, the duration of changes in BBB function after LiFUS exposure, the extent of BBB modulation, and dose-safety studies for the therapeutics administered [12,13,14]. However, the fundamental questions regarding the potential impact of LiFUS-mediated BBB disruption on multiple aspects of neurophysiology and the relationship between these physiological changes and therapeutic benefits remain unanswered [15]. Preliminary studies of the secondary effects of LiFUS suggest roles for inflammatory responses, proteomic and transcriptomic changes, alterations in cerebral blood flow, modulation of neuronal activity, and clearance of metabolic waste products [15,16,17]. To date, the understanding of changes in adjacent non-sonicated tissues and the timing of BBB endothelial repair remains limited. Such knowledge is crucial with the extension of focused ultrasound to clinical trials for a broad range of applications, including disruption, neuromodulation, and ablation.

We have previously demonstrated that, in addition to an immediate increase in BBB permeability following LiFUS exposure, a secondary increase in BBB permeability occurs 6 h post LiFUS [12]. This pattern of biphasic BBB opening has previously been reported in stroke and traumatic brain injury. One hypothesized mechanism underlying biphasic BBB opening is the rupture and subsequent widespread repair of tight junctions and transporter proteins that maintain BBB integrity [18]. Additional hypotheses suggest the recruitment of repair-associated biological processes, including cellular inflammation, innate and adaptive immunity, and stress responses [15]. While most of these responses are associated with detrimental inflammatory reactions, they can also lead to cell repair, differentiation, and neurogenesis [19,20,21,22,23,24].

This study aimed to examine the relationship between LiFUS-induced increases in BBB permeability and changes in BBB architecture, as well as the effects of LiFUS on non-sonicated adjacent tissues. In this study, we utilized in situ brain perfusions to evaluate changes in the function and expression of essential BBB transporters and structural proteins after exposure to LiFUS, in addition to changes in sterile inflammatory response post LiFUS, by MSD analysis.

## 2. Materials and Methods

### 2.1. Chemicals

The components for the physiologic buffer, including NaCl, NaHCO_3_, KCl, NaH_2_PO_4_, CaCl_2_, MgSO_4_, and D-glucose, were all obtained from Sigma (San Jose, CA, USA). Meanwhile, ^14^C sucrose, ^3^H-glucose, and ^3^H-Ivermectin were obtained from Moravek Biochemicals Inc. (Brea, CA, USA). Cytokine analysis was performed using the VPLEX Proinflammatory Panel 1 purchased from mesoscale discovery (Rockville, MD, USA).

### 2.2. Animals

All in vivo experiments were carried out in compliance with the National Institutes of Health’s Guide for the Care and Use of Laboratory Animals and were approved by the Institutional Animal Care and Use Committee at the West Virginia University. Female mice (athymic Nu/Nu or BALB/cJ) were purchased from the Jackson Laboratory (Bar Harbor, ME, USA). Treatments were performed when the mice were 4–6 weeks old and weighed approximately 25 g. The animals were allowed to acclimate for at least 1 week before the experiments.

### 2.3. Low-Intensity Focused Ultrasound Treatment

LiFUS treatments and targeting were carried out using the ExAblate Neuro 4000 ultrasound technology (InSightec, Haifa, Israel), as previously described [12]. Briefly, a T1-weighted turbo-spin echo sequence was used to target in the coronal, sagittal, and axial planes. Consistent positioning and the adjustment of animals on the ultrasound transducer were ensured by the use of a plastic frame constraint. The baseplate and rodent bed assembly allowed mice to be placed in a supine position with their heads half submerged in degassed water. Based on animal size and weight, the animal bed featured dials for both vertical and horizontal adjustment. The one-channel 3 inch MRI coil and specially constructed nose seal ensured the stationary placement of the assembly at the MRI isocenter. Heating pads were used to maintain the mice’s body temperatures throughout the experiment. During all the experiments, the animals were anesthetized using 1–2% isoflurane and examined closely for any indications of distress. An intravenous injection of Definity^®^ microbubbles (Lantheus Imaging) at a dose of 40 μL/kg was administered immediately before sonication. The mice were sonicated 10–20 s after microbubble injection. The sonications were performed at 0.3 cavitation dose for 60 s based on our previous data. A gradient echo/flash-weighted sequence was run to capture local macro-hemorrhage post sonication.

### 2.4. Determination of Vascular Volume, Glucose, and P-gp Function by In Situ Brain Perfusion

Vascular permeability and glucose and P-gp transporter function were evaluated by determining 14-C sucrose, ^3^H glucose, and ^3^H Ivermectin uptake using the in situ brain perfusion technique of Takasato et al., with modifications [25,26]. The perfusions were performed using a Tris-buffered physiological perfusate containing NaH_2_PO_4_ (2.4 mM), KCl (4.2 mM), NaHCO_3_ (24 mM), NaCl (128 mM), CaCl_2_ (1.5 mM), MgCl_2_ (0.9 mM), and D-glucose (9 mM). The perfusion fluid was vortexed, filtered, and warmed to 37 °C before use. The mice were anesthetized using xylazine (6–8 mg/kg) and ketamine (75–100 mg/kg) and checked for adequate sedation using a toe-pinch. The chest cavity of the mice was exposed, followed by clamping the descending aorta using a microclamp and an applicator. Blood recirculation was prevented by making an incision in the right atrium. A PE-50 catheter was placed in the left cardiac ventricle using a butterfly syringe, followed by perfusion for 15–120 s using an infusion pump at 5 mL/min (Harvard Apparatus, Holliston, MA, USA). Immediately after the perfusion, the mice were decapitated with the rapid removal of the brain. The meningeal vessels and arachnoid membrane were removed, and the brain was dissected on ice. Each region was individually weighed and digested overnight in 1 mL Solvable (PerkinElmer, Waltham, MA, USA) at 50 °C. The next day, the samples were allowed to equilibrate at room temperature, followed by the addition of 10 mL Ultima-Gold Scintillation fluid (PerkinElmer, MA, USA). The samples were analyzed using dual-labeled PerkinElmer TriCarb 4910 liquid scintillation counting (Waltham, MA, USA). The concentration in the brain (Q*) and perfusate (C*) was expressed as DPM/g or DPM/mL. The vascular volume Vo (mL/g) and the unidirectional uptake transfer coefficient Kin (mL/s/g) were calculated as previously described, using the following equations [25,27]:

Brain distribution volume = Q*/C*

Q*/C* = Kin(T) + Vo

Vo refers to the initial uptake rate of the compound in the brain, derived from the slope of the early linear phase vs. the plasma AUC. This early phase represents the time when transport across the blood–brain barrier is not significantly influenced by metabolism or other secondary processes.

### 2.5. Isolation of Brain Capillaries

Brain capillaries from the mice were isolated in a modified protocol, as previously stated in [28]. Briefly, the mice were euthanized, and their brains were removed and sectioned into sonicated and non-sonicated hemispheres immediately after sonication or at predetermined time-points (6 h, 12 h, and 24 h). The mice brains were transferred to a Dounce tissue homogenizer with 15 mL of isolation buffer consisting of PBS with 5 mM of D-glucose and 1 mM of sodium pyruvate. The brains were homogenized carefully, transferred to a 50 mL conical tube, and centrifuged at 4 °C at 2000× *g* for 10 min. The pellet was re-suspended in an 18% dextran gradient solution and centrifuged at 4 °C at 4255× *g* for 17 min. The supernatant, including the myelin layer, was removed, and the pellet was suspended in 5 mL of 1% BSA in PBS. The suspension was first filtered through a 100 µm filter then through a 20 µm filter. The capillaries collected on the filter were washed in 1% BSA in PBS and centrifuged at 4 °C at 1500× *g* for 5 min. The final capillary pellet was checked under an inverted microscope and stored in aliquots at −20 °C until use.

### 2.6. Multiplex Pro-Inflammatory Panel for the Determination of Brain Cytokine Levels

Mouse brains were collected and processed for cytokine analysis as previously described [29]. Briefly, brain tissue was collected, dissected, and stored in RNA (Invitrogen, Waltham, MA, USA) until processing. The protein content was evaluated by homogenization of tissue sections in 1 mL/100 mg of RIPA Buffer (Thermo Fisher Scientific, Waltham, MA, USA) containing a Halt Protease and Phosphatase Inhibitor Cocktail (Thermo Fisher Scientific, MA, USA) by sonication. After 30 min of incubation on ice, the samples were centrifuged at 4 °C at 13,300 rpm for 15 min. The supernatant was collected and evaluated for protein content using the Bradford Assay. The cytokine concentrations within the brain and serum samples were evaluated using the V-Plex Pro-inflammatory Mouse 10-spot 96-well plate based on the manufacturer’s protocol. The calibrators, prepared samples, and controls were added to the plate and incubated overnight with shaking. After washing the plates, the antibodies detected for IFNγ, IL-10, IL-1β, IL-2, TNFα, IL-4, IL-5, IL-6, KC/Gro (CXCL1), and IL-12 p70 were added and incubated for 2 h at room temperature with constant shaking. The plates were then washed, followed by the addition of 2× Read Buffer before reading each plate on a Meso Quickplex SQ120 plate reader. The calibrator standard curve was used to calculate the pg/mL concentration for each cytokine.

### 2.7. Statistical Analysis

The evaluation of differences between groups for cytokine analysis and in situ perfusions were performed using one-way ANOVA or an unpaired two-tailed Student’s T test followed by a Bonferroni correction. For all the experiments, the errors listed in this paper are the standard error of the mean (SEM), unless indicated otherwise (n = 5–8). Differences were considered statistically significant at *p* < 0.05 (*) and *p* < 0.01 (**). The assessment of statistical significance between the treatment groups was carried out using GraphPad^®^ Prism 7.05 (San Diego, CA, USA).

## 3. Results

### 3.1. LiFUS Increases Cerebral Vascular Volume and Glucose Uptake 6 h Post Sonication

**Figure 1:**
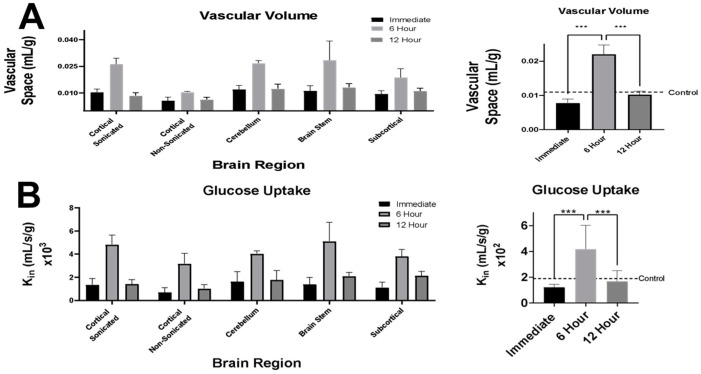
Vascular and glucose uptake changes 6 h post LIFU. Vascular volume (**A**) and glucose uptake (**B**) were significantly increased across the whole brain and within regional distributions (*p* < 0.01). The error bars represent the standard error of the mean (SEM). Statistical significance is indicated by an asterisk (***).

The vascular volume (V_v_) within the control and experimental groups was assessed using ^14^C sucrose, a vascularly impermeable marker of BBB integrity. In all the treated groups, an increase in vascular volume suggested a disruption of BBB integrity, as observed in Figure 1A. LiFUS induced a significant increase in V_v_ (2.20 ± 0.12 × 10^−2^ mL/g) 6 h following sonication, with V_v_ (1.023 ± 0.39 × 10^−2^ mL/g) not significantly differing from the control at 12 h post sonication. Regional differences in BBB permeability were observed 6 h post sonication, which did not persist at the 12 h time-point.

We also measured changes in cerebral ^3H^Glucose uptake after LiFUS-mediated BBB disruption, as shown in Figure 1B. The uptake of ^3H^Glucose in healthy non-sonicated mouse brain tissue was 1.93 ± 0.92 × 10^−2^ mL/s/g. LiFUS did not induce a significant change in ^3H^Glucose uptake immediately following sonication (1.22 ± 1.002 × 10^−2^ mL/s/g), but it significantly increased the uptake of ^3H^Glucose 6 h after sonication (4.187 ± 1.84 × 10^−2^ mL/s/g). ^3H^Glucose uptake 12 h post sonication (1.68 ± 0.84 × 10^−2^ mL/s/g) was not significantly different from the control. ^3H^Glucose uptake did not significantly differ between brain tissue regions at any time-point post LiFUS treatment.

### 3.2. LiFUS Reduces P-Glycoprotein Function 6 h Post Sonication

**Figure 2:**
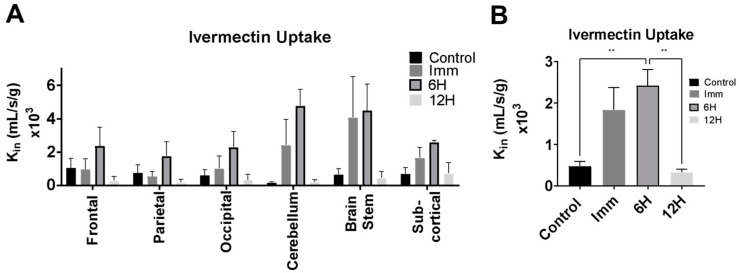
Reduction in P-glycoprotein (P-gp) function 6 h post LIFU. P-gp activity showed a significant decrease in both regional distribution (**A**) and total brain analyses (**B**) (*p* < 0.01). The error bars represent the standard error of the mean (SEM). Statistical significance is denoted by an asterisk (**).

P-glycoprotein (P-gp) function post LiFUS was evaluated by measuring the uptake of ^3H^Ivermectin, a known P-gp substrate, as shown in Figure 2. ^3H^Ivermectin uptake was significantly increased 6 h after LiFUS (2.42 ± 0.38 × 10^−3^ mL/s/g) compared to the control non-sonicated mice (0.47 ± 0.11 × 10^−3^ mL/s/g), but it was not significantly different from the control neither immediately following sonication (1.83 ± 0.54 × 10^−3^ mL/s/g) nor at 12 h post sonication (0.33 ± 0.07 × 10^−3^ mL/s/g). ^3H^Ivermectin uptake did not differ significantly between brain tissue regions at any time-point post LiFUS.

### 3.3. LiFUS Induces Pro-Inflammatory Cytokine Release in Sonicated and Non-Sonicated Tissues

**Figure 3:**
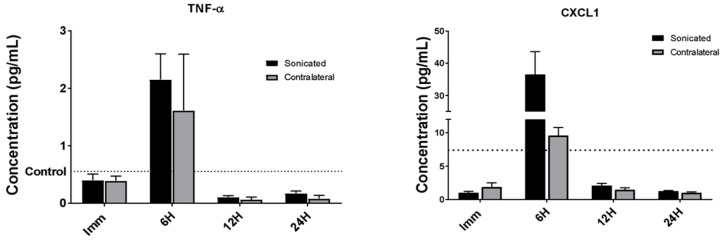

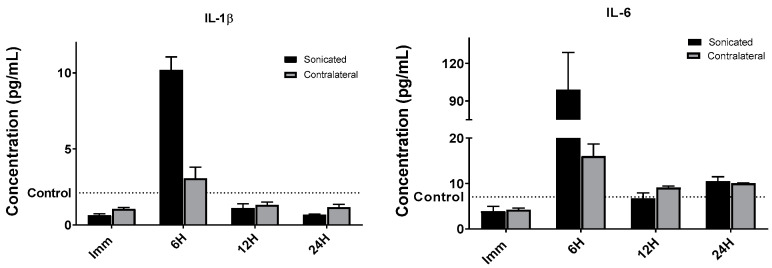
Temporal changes in pro-inflammatory cytokine expression post LIFU. The levels of TNF-α, CXCL1, IL-1β, and IL-6 were significantly elevated in the sonicated and contralateral brain regions after 6 h and returned to the baseline at 12 h post sonication (*p* < 0.01). No significant changes were observed for IL-5, IL-10, IFN-γ, and IL-2 at any of the evaluated time-points.

We examined the production of cytokines to evaluate the extent and timing of immune responses post LiFUS. As shown in Figure 3, the expression of TNF-α, CXCL1, IL-1β, and IL-6 was significantly increased within the sonicated region compared to the brain tissue of untreated control mice 6 h post LiFUS. The expression of TNF-α was also significantly increased in the contralateral hemisphere after 6 h compared to the control, while the contralateral increases in CXCL1, IL-1β, and IL-6 expression were not significant. No significant differences were found in the concentrations of IL-2, IFN-γ, IL-10, IL-4, IL-5, and IL-12p70 between the control, sonicated, and contralateral brain regions at any of the evaluated time-points.

**Figure 4:**
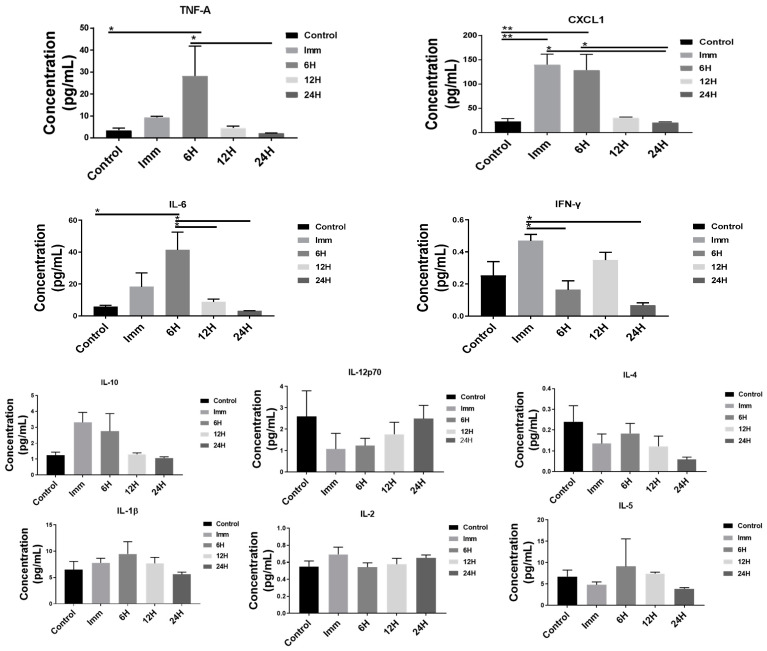
Serum cytokine expression following LIFU: TNF-α, CXCL1, IL-6, and IFN-γ were significantly increased in the serum 6 h post sonication (*p* < 0.01). No significant differences were observed for IL-10, IL-12p70, IL-4, IL-1β, IL-2, or IL-5 in the serum. Statistical significance is denoted by an asterisk (*, **).

The concentrations of TNF-α, CXCL1, and IL-6 were found to be significantly higher in the serum after 6 h compared to the control, as shown in Figure 4. The levels of IFN-γ were significantly higher immediately after sonication in the serum compared to the 6 h and 24 h groups. Similarly, the serum expression of CXCL1 was higher immediately after sonication compared to the control and 24 h groups. All changes returned to the baseline 12 h post sonication. No significant changes in the serum concentrations of IL-2, IL-10, IL-4, IL-5, IL-1β, and IL-12p70 were found between 0 and 24 h post sonication.

**Figure 5:**
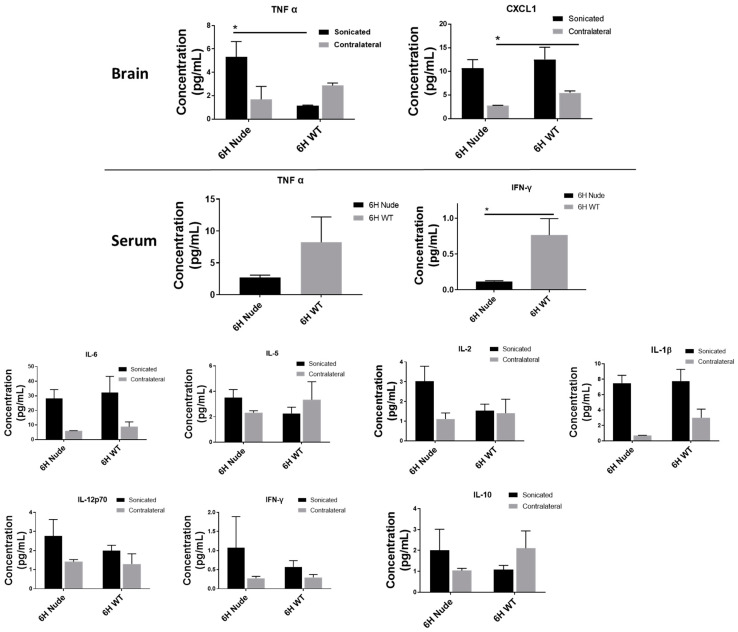

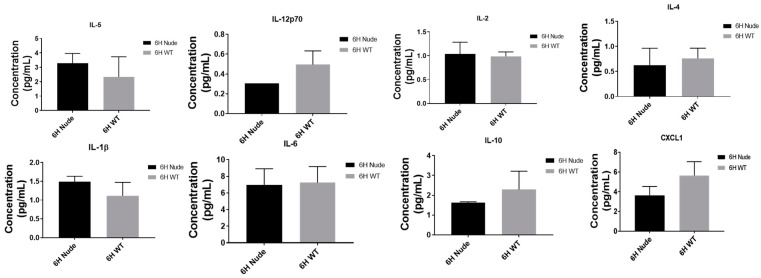
Differential cytokine response in wild-type and immunocompromised mice post LIFU: TNF-α and CXCL1 exhibited significant differences in the sonicated and contralateral brain after 6 h in both wild-type (Balb/c) and immunocompromised (Nu/Nu) mice (*p* < 0.01). In the serum, the TNF-α and IFN-γ levels were also significantly lowered. Some cytokines showed no significant differences between groups in both brain and serum analyses. Statistical significance is denoted by an asterisk (*).

Next, we evaluated changes in the brain and serum concentration of pro-inflammatory cytokines after 6 h in wild-type BALB/c and Nu/Nu mice, as shown in Figure 5. The serum concentrations of IFN-γ and TNF-α were significantly lower in Nu/Nu mice compared to the Balb/c wild-type, as shown in Figure 5. While the concentration of TNF-α was significantly higher in sonicated Balb/c wild-type brains compared to the Nu/Nu sonicated and contralateral regions, the expression of CXCL-1 was significantly higher in the contralateral Nu/Nu region compared to Balb/c wild-type species, as shown in Figure 5. No significant changes in the serum concentrations of IL-2, IL-10, IL-4, IL-5, IL6, CXCL1, and IL-1β were found after 6 h between the two strains. Similarly, there were no significant changes in the sonicated and contralateral brain concentrations of IL-2, IFN-γ, IL-5, IL6, IL-1β, IL-12p70, and IL-10 after 6 h between Balb/c wild-type and Nu/Nu mice. The serum concentration of IL-12p70 in Balb/c mice and the brain concentration of IL-4 failed to reach the detectable range.

## 4. Discussion

In the past few years, BBB disruption using LiFUS has emerged as a procedure to increase drug efficacy in the CNS. A majority of early studies on LiFUS BBB modulation have concentrated on establishing the ideal parameters for higher therapeutic efficacy and safety, as with any new medical device [7,30]. Extensive preclinical research on BBB opening by LiFUS has led to the utilization of LiFUS in clinical trials for a variety of CNS diseases [31,32,33,34,35]. Although there are not many documented concerns with immediate LiFUS exposure, little is known regarding the secondary downstream effects at the BBB after short- and long-term LiFUS use [36,37,38]. Further, BBB breakdown in CNS disorders such as stroke and traumatic brain injury are known to elicit neurophysiological responses that may exacerbate the underlying condition [39,40,41,42]. The mechanism of LiFUS-associated BBB opening is hypothesized to be due to a primary compression and oscillation of the microbubbles at the BBB lining, followed by a secondary reaction which occurs hours after the primary exposure [15,42]. Herein, we investigated the relationship between increased LiFUS-mediated BBB permeability and the window of secondary changes in sonicated and contralateral BBB post sonication.

First, we investigated changes in vascular volume and glucose uptake at different time-intervals post LiFUS. We used an in situ brain perfusion technique to determine unidirectional glucose transport kinetics. This approach utilized a known perfusion medium at a controlled rate and time-interval to study the rate of substrate influx without systemic interference [27,43]. Consistent with our previous findings, we observed an increase in the permeability of our passive marker in the cortical regions immediately after sonication [12]. Further, there was a secondary increase in permeability 6 h after sonication, which returned to the baseline at 12 h post LiFUS. Previous research has shown that high glucose with persistent hyperglycemia can appear as a complication in patients with traumatic brain injury [44]. These patients often show poor disease prognosis. We observed an increase in the function of GLUT-1 transporters, as demonstrated by the increased uptake of ^3^H-Glucose at 6 h post sonication. It is possible that the increased glucose requirement is related to an enhanced repair-associated energy demand by neurons, astrocytes, and endothelial cells at the BBB. The increase in vascular volume and glucose uptake suggests a delayed and biphasic opening of the BBB, similar to that observed in stroke and TBI after insult to the capillaries within the brain.

Next, we measured ^3^H-Ivermectin uptake to evaluate P-gp function at the BBB post LiFUS. Our in situ brain perfusion technique revealed that the accumulation of 3H-Ivermectin within the brain was significantly higher after 6 h compared to the control and 24 h groups. While there was an increase in 3H-Ivermectin uptake immediately after sonication, it was not significantly different than the other groups. These data suggest that there is a slow decline in the function of P-gp immediately after LiFUS and that this decline peaks at 6 h after sonication. The rate of P-gp function returns to the baseline at 24 h post sonication. While P-gp is essential for eliminating potentially toxic chemicals and compounds in a healthy BBB, it can also effectively prevent the accumulation of CNS active agents at therapeutically effective concentrations [45,46]. Many efforts have studied the inhibition of P-gp for increased therapeutic delivery for neurodegenerative diseases and brain tumors, with limited clinical success [47,48]. Selective and transient reduction in P-gp function may be exploited as a superior means to increase drug accumulation across the generally impenetrable BBB.

We then evaluated differences in the expression of pro-inflammatory cytokines within the sonicated and contralateral regions of the brain. We observed a peak in the expression of TNF-A, CXCL1, IL-1b, and IL-6 in the sonicated brain 6 h after LiFUS. A higher expression of TNF-A in the contralateral brain was also observed at the 6 h time-point. Although CXCL1, IL-1b, and IL-6 expression in the contralateral area increased after 6 h, it did not differ substantially from the control animals. The serum concentrations of TNF-A, CXCL1, and IL-6 were significantly greater after 6 h. However, IFN-γ and CXCL1 expression in the serum was found to be significantly higher immediately after sonication. All changes returned to the baseline at 12 h post sonication, suggesting the diminishing of the inflammatory response with the repair of the BBB endothelium. The activation of sterile inflammatory pathway can be beneficial and lead to downstream repair and cell growth, or it might worsen the underlying pathology [15,49,50]. Previous reports suggest that neurogenesis can be activated in response to the inertial cavitation associated with LiFUS BBB opening. The activation of IL-1b, IL-6, and TNF-A after 6 h in our experiments supports these findings [51,52,53].

Differences in the concentration of pro-inflammatory cytokines in the brain and serum of wild-type BALB/c and Nu/Nu mice were then assessed at the 6 h time-point. Immunodeficient Nu/NU mice had significantly lower serum IFN-g and TNF-a than Balb/c wild-type mice. However, TNF- α was higher in the sonicated Balb/c wild-type brain compared to the Nu/Nu sonicated and contralateral regions. The expression of CXCL1 in the Nu/Nu contralateral region was higher than in the Balb/c contralateral region. A prior study investigating the amount and role of T cell-associated cytokines (IL-2, IL-1b, IFN-g, TNF-a) in nude and wild-type Alzheimer’s mice discovered that neuronal cell regeneration was dependent on cytokine expression [54]. They found that the administration of Aβ1–42 peptide produced lower peripheral serum IFN-γ in BALB/c-nude mice than in wild-type mice, which correlated with higher hippocampal neuronal regeneration in the wild-type mice [54]. The study also showed a higher expression of TNF-a in the wild-type Alzheimer’s mice compared to the T cell-deficient nude mice [31,37,54]. Another study showed that acute inflammation triggered stronger innate immune reactions in immunosuppressed mice compared to the wild-type variant [55]. These data correlate with our current study and suggest a deeper investigation into the correlation between cytokine expression and neuronal regeneration.

While our study expands on previous research within the field, there are a few limitations. First, we demonstrated an increase in functioning of 3^H^ Glut-1 and P-gp; however changes in their expression post LIFUS need to be evaluated. The initial increase in Ivermectin concentrations following sonication may result from a combination of potential alterations in P-gp function and enhanced diffusion due to BBB disruption. This could be addressed in future research through competitive inhibition studies to assess P-gp activity. Additional changes in genomic expression within the sonicated and contralateral regions also need to be evaluated by rt-PCR and genomic analysis. Moreover, the function and expression of endothelial proteins like JAMs, OATPs, LATs, etc., need to be further evaluated. There is also a need to delineate the extent of transporter inhibition by the competitive inhibition of passive transport via the addition of unlabeled substrate. Lastly, the relationship between pro-inflammatory cytokine production and LiFUS BBB opening needs to be investigated further to understand potential side-effects, as well as repair and cell regeneration mechanisms.

## 5. Conclusions

LiFUS is a non-invasive technique for transient and targeted BBB opening. Over the past several years, there has been an increase in studies concerning the secondary consequences to transcranial BBB opening by LiFUS. There is a need to understand the acute and chronic effects on the brain’s neurophysiology with increased LiFUS-induced BBB permeability. This work highlights a time-dependent change in the BBB microenvironment on the sonicated and contralateral sides post LiFUS. We also demonstrate the window of heightened inflammatory responses to LiFUS and offer a potential mechanism for further exploration. These findings underscore the need to consider both therapeutic and secondary effects, such as inflammation and BBB repair, in the application of LiFUS for CNS drug delivery. These findings have significant implications for the clinical use of LiFUS, particularly in optimizing treatment parameters to maximize therapeutic efficacy through timed drug delivery during periods of reduced P-gp activity or maximal BBB permeability, while minimizing adverse effects.

## Data Availability

The original contributions presented in this study are included in the article; further inquiries can be directed to the corresponding author/s.
